# Baseline Assessment of WOAH-Listed Protozoan Parasites in Wild Mediterranean Mussels *Mytilus galloprovincialis* and Pacific Oysters *Crassostrea gigas* from Port-Adjacent Coastal Waters of Korea in 2023

**DOI:** 10.3390/ani16101502

**Published:** 2026-05-14

**Authors:** Jeong-Hwa Kim, Nobuhisa Kajino, Jong-Seop Shin, Hee Jung Choi, Mun-Gyeong Kwon, Chan-Il Park, Kwang-Sik Choi, Hyun-Ki Hong

**Affiliations:** 1 Department of Marine Life Science (BK21 FOUR) and Marine Science Institute, Jeju National University, Jeju 63243, Republic of Korea; star0798@jejunu.ac.kr (J.-H.K.);; 2 Tropical & Subtropical Research Center, Korea Institute of Ocean Science and Technology (KIOST), Jeju 63349, Republic of Korea; 3 Aquatic Disease Control Division, National Fishery Products Quality Management Service, Busan 46083, Republic of Korea; 4 Department of Marine Biology and Aquaculture, Gyeongsang National University, Tongyeong 53064, Republic of Korea

**Keywords:** *Bonamia*, harbor, *Marteilia*, Mediterranean mussel, Pacific oyster, *Perkinsus*

## Abstract

Systematic surveillance of World Organization for Animal Health (WOAH)-listed protozoan parasites is essential for maintaining the sanitary status of seafood exports and detecting the introduction of exotic pathogens into coastal ecosystems. In 2023, we examined wild Mediterranean mussels *Mytilus galloprovincialis* and Pacific oysters *Crassostrea gigas* collected from small harbors adjacent to ten major trading ports along the west and south coasts of Korea to assess the occurrence of WOAH-listed protozoan parasites and emerging *Perkinsus* species. A total of 1080 mussels and 1080 oysters from 18 sites were sampled in spring and autumn, and gill and digestive gland tissues were pooled from six individuals for DNA extraction. Species-specific PCR assays targeting *Perkinsus marinus*, *P. olseni*, *P. beihaiensis*, *Bonamia ostreae*, *B. exitiosa*, and *Marteilia refringens* were performed using previously validated primer sets and positive controls. All PCR assays were negative for the six protozoan parasite species in both host species across all sampling sites, indicating no detectable infections in port-adjacent wild mussel and oyster populations during the survey period. These negative results contrast with recent reports of *P. marinus* in wild *C. gigas* and *B. ostreae* in *Ostrea denselamellosa* on the west coast of Korea, suggesting that infections may currently be focal, transient, and host-specific rather than widespread in port-associated *M. galloprovincialis* and *C. gigas* populations. The present study provides baseline data on the distribution of protozoan parasites in bivalves inhabiting high-risk harbor environments and underscores the need for continued surveillance of transboundary shellfish diseases that is closely coupled with environmental monitoring.

## Simple Summary

Wild mussels (*Mytilus galloprovincialis*) and oysters (*Crassostrea gigas*) collected near major Korean ports in 2023 were screened for six WOAH-listed protozoan parasites, including *Perkinsus*, *Bonamia*, and *Marteilia* species using PCR. No infections were detected in any samples, indicating an absence of these pathogens in port-adjacent populations during the study period. However, recent localized reports in other hosts suggest limited, host-specific occurrence. These results provide baseline data and support the need for continued surveillance in high-risk coastal areas.

## 1. Introduction

In the international seafood trade, importing countries implement sanitary and phytosanitary measures in line with the World Organization for Animal Health (WOAH) guidelines to protect their aquatic ecosystems and aquaculture industries [1]. Scientific surveillance data demonstrating that exported products are free from WOAH-listed aquatic animal diseases are a key requirement for meeting import sanitary standards [2,3]. Bivalves, due to their sessile lifestyle and filter-feeding behavior, are widely used as sentinel species that reflect environmental conditions and the presence of pathogens in coastal ecosystems [4,5]. Evidence of pathogen dispersal through maritime transport has been suggested by detections of *Perkinsus beihaiensis* infections in Mediterranean mussels *Mytilus galloprovincialis* in Tokyo Bay, Japan, and in bivalve populations in Panama [6,7,8].

Marine protozoan parasites infecting bivalves differ markedly in their phylogenetic position, tissue tropism, and transmission strategies. *Perkinsus* spp. are alveolate parasites belonging to Perkinsozoa, a lineage phylogenetically positioned between dinoflagellates and apicomplexans, and their relatively direct host-to-host life cycle, in which multiple life stages can be infective, may facilitate their spread through infected molluscs and contaminated environments [9]. *Perkinsus* species, the causative agents of perkinsosis, are known to occur as trophozoites within host tissues, whereas in the surrounding environment, including seawater and sediments, they may persist in other life-history stages, such as hypnospores [10,11]. Similarly, *Bonamia* spp. are haplosporidian protozoan parasites that primarily infect oyster haemocytes, and direct transmission without a confirmed intermediate host has been suggested, allowing systemic infection and mortality in susceptible flat oyster hosts [12,13]. In contrast, *Marteilia refringens*, a member of the Paramyxea/Marteiliidae, mainly infects the digestive tract of bivalves, with early stages occurring in the epithelium of the palps, oesophagus, and stomach, and sporulation occurring in the digestive gland [14,15,16]. Unlike *Perkinsus* and *Bonamia*, *M. refringens* is thought to have an indirect and more complex life cycle involving planktonic copepods such as Paracartia grani. Therefore, the absence or low abundance of suitable intermediate hosts in a given ecosystem may act as a natural barrier limiting parasite transmission [17].

The Mediterranean mussel *M. galloprovincialis* is a well-recognized invasive species [18,19,20] that can be infected by *M. refringens*, *P. olseni*, and *P. beihaiensis*, indicating its susceptibility to a range of protozoan parasites affecting bivalves [6,14,21,22,23]. The Pacific oyster *Crassostrea gigas* is susceptible to *P. marinus* and has also been suggested as a potential vector of *Bonamia ostreae* [12,13,24,25,26]. These protozoan parasites are of particular concern because they can cause significant mortality and productivity loss in wild and farmed bivalves and may be translocated between regions through the movement of seed, live shellfish, and shipping activities [27]. Along the Korean coast, *P. olseni* is known to be prevalent in Manila clams and blood cockles [28], and previous PCR-based monitoring reported no WOAH-listed protozoan parasites in *C. gigas* along parts of the southern coast [29]. More recently, however, *P. marinus* was detected in wild *C. gigas* on the west coast of Korea, and *B. ostreae* infection was reported in wild flat oysters *Ostrea denselamellosa* near Gunsan Port [25,30], raising concerns about the introduction and local establishment of these exotic protozoan parasites. Among the targeted protozoan parasites, *P. marinus*, *P. olseni*, *Bonamia* spp., and *M. refringens* are WOAH-listed, whereas *P. beihaiensis* is a non-listed but emerging *Perkinsus* species with documented occurrences in bivalves from Japan and Panama [6,7,8].

Although most surveillance efforts for protozoan parasites in Korea have focused on oysters, wild mussel populations may also represent important hosts or sentinels for parasite occurrence in coastal environments. In particular, *M. galloprovincialis* has been reported as a host susceptible to infection by *M. refringens* in other regions [31], suggesting that mussel populations in Korea should not be overlooked in surveillance programs. However, information on the occurrence of target protozoan parasites and emerging *Perkinsus* species in wild *M. galloprovincialis* inhabiting port-adjacent waters of Korea remains limited. This represents an important knowledge gap, because port-adjacent environments are potential entry points for non-native pathogens associated with vessel traffic, biofouling communities, ballast or exchanged seawater, and the movement of aquatic organisms.

Therefore, this study aimed to investigate the occurrence of the major protozoan parasites *P. marinus*, *P. olseni*, *B. ostreae*, *B. exitiosa*, and *M. refringens* in wild bivalves, namely *M. galloprovincialis* and *C. gigas*, using molecular diagnostic methods, and to assess the potential introduction or distribution of these pathogens in Korea.

## 2. Materials and Methods

### 2.1. Sample Collection

**Figure 1:**
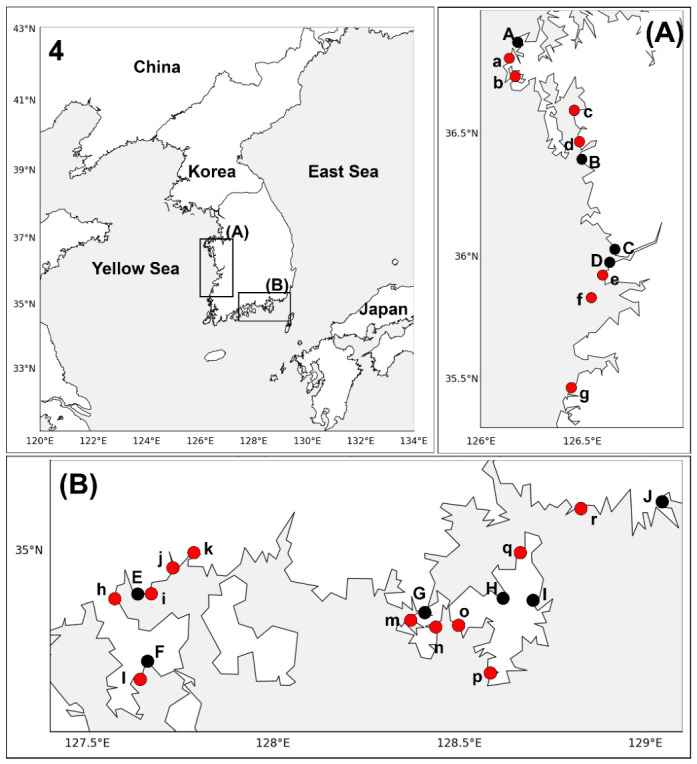
Map showing sampling locations of *Mytilus galloprovincialis* along the west (**A**) and south coasts (**B**) of Korea. A, Taean Port; B, Boryeong Port; C, Janghang Port; D, Gunsan Port; E, Gwangyang Port; F, Yeosu Port; G, Tongyeong Port; H, Gohyeon Port; I, Okpo Port; J, Busan Port; a, Eoeundol Port; b, Tonggae Port; c, Gungri; d, Namdang Port; e, Bieung Port; f, Jangja-do; g, Gusipo Port; h, Johwa-ri; i, Gwangyang Police Station; j, Dochon Ferry Terminal; k, Mangdeok Port; l, Hodu-ri; m, Pyeongrim Port; n, Hwasam-ri; o, Eogu-ri; p, Myeongsa-Port; q, Jangmok-ri; r, Angolpo.

**Figure 2:**
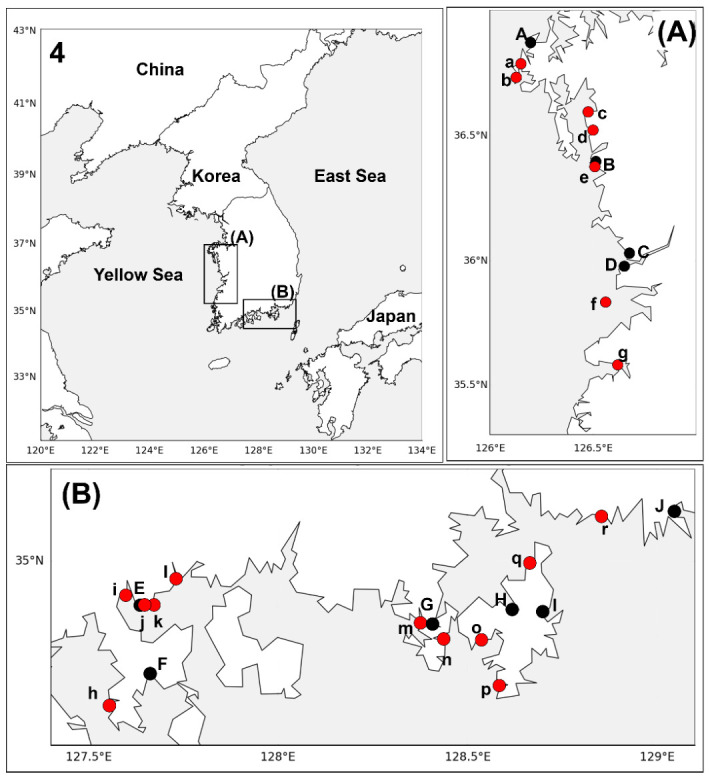
Map showing sampling locations of *Crassostrea gigas* along the west (**A**) and south coasts (**B**) of Korea. A, Taean Port; B, Boryeong Port; C, Janghang Port; D, Gunsan Port; E, Gwangyang Port; F, Yeosu Port; G, Tongyeong Port; H, Gohyeon Port; I, Okpo Port; J, Busan Port; a, Mohang Port; b, Pado-ri; c, Gungri; d, Suryong Port; e, Janghang Port; f, Seonyu Port; g, Gomso Port; h, Imok-ri; i, Chonam Bridge; j, Gwangyang Port; k, Gwangyang Police Station; l, Taein Bridge; m, Pyeongrim Port; n, Dongam Port; o, Seondaldo Ferry Terminal; p, Daepo Port; q, Jangmok-ri; r, Busan New Port.

To evaluate the occurrence of WOAH-notifiable protozoan parasites in port-associated bivalve populations, wild *M. galloprovincialis* and *C. gigas* were collected from small harbors near ten major trading ports along the west and south coasts of Korea. The samples were then screened using species-specific PCR assays for *P. marinus*, *P. olseni*, *P. beihaiensis*, *B. ostreae*, *B. exitiosa*, and *M. refringens*. In May and September 2023, mussels and oysters were sampled from 18 sites around Taean, Boryeong, Janghang, and Gunsan on the west coast and Gwangyang, Yeosu, Tongyeong, Gohyeon, Okpo, and Busan on the south coast (Figure 1 and Figure 2), yielding a total of 1080 mussels and 1080 oysters for analysis. Shell height (for mussels) or shell length (for oysters) and tissue weight were measured for each individual, and the data were expressed as the mean ± standard deviation for each sampling site and sampling period. Gill and digestive gland tissues were excised. The digestive gland was defined as the dark brown digestive diverticula in the visceral mass, consisting of digestive tubules/acini and associated ducts connected to the stomach, while the stomach and intestine were excluded during dissection. The tissues were stored at −70 °C for molecular analysis and remaining tissues preserved in Davidson’s solution (glycerin 1.37 M, formaldehyde 2.67 M, ethanol 5.09 M, acetic acid 1.75 M, filtered seawater 300 mL/L) for subsequent histological observation following confirmation of PCR positivity.

### 2.2. PCR Assay

For PCR screening, approximately 30 mg of pooled gill and digestive gland tissues, digestive tubules/acini, and associated ducts connected to the stomach from six individuals were combined to form a single sample, and genomic DNA was extracted using the DNeasy Blood & Tissue Kit (Qiagen, Hilden, Germany) following proteinase K digestion, resulting in pooled DNA samples that were used as templates for species-specific PCR assays. This pooling strategy was used as a first-line surveillance approach to increase the spatial and sample coverage of the survey while keeping the number of DNA extractions and PCR reactions manageable. Six primer sets targeting WOAH-listed and non-listed protozoan parasites, *P. marinus*, *P. olseni*, *P. beihaiensis*, *B. ostreae*, *B. exitiosa*, and *M. refringens*, were selected based on previously published species-specific PCR assays for each target species (Table 1). The primer sequences, target regions, expected amplicon sizes, and references are summarized in Table 1. All primers were commercially synthesized by Macrogen Inc. (Seoul, Republic of Korea). Each PCR reaction was performed in a 25 μL volume containing 100 ng of template DNA, buffer with MgCl_2_, dNTPs, primers, and Ex Taq DNA polymerase (Takara, Kusatsu, Japan), using thermal cycling conditions based on the original primer descriptions with minor modifications. For *P. marinus* and *P. olseni*, DNA extracted from in vitro cultures was used as a positive control, whereas plasmid DNA of *B. ostreae*, *B. exitiosa*, and *M. refringens* from the European Union Reference Laboratory for Mollusk Diseases and plasmid DNA of *P. beihaiensis* provided by the University of Tokyo served as positive controls for these targets. PCR products were separated on 1.5% agarose gels along with a 100 bp DNA ladder and visualized under UV illumination to detect specific amplicons corresponding to each protozoan parasite. The pooled-sample positivity rate was calculated as the percentage of PCR-positive pooled samples among all pooled samples analyzed. As each pooled sample consisted of tissues from six individuals, this value does not represent individual-level infection prevalence and should be interpreted as a pooled-sample screening result. A limitation of this approach is that low-intensity infections in individual animals may be diluted within pooled samples.

## 3. Results

### PCR Screening Results

**Figure 3:**
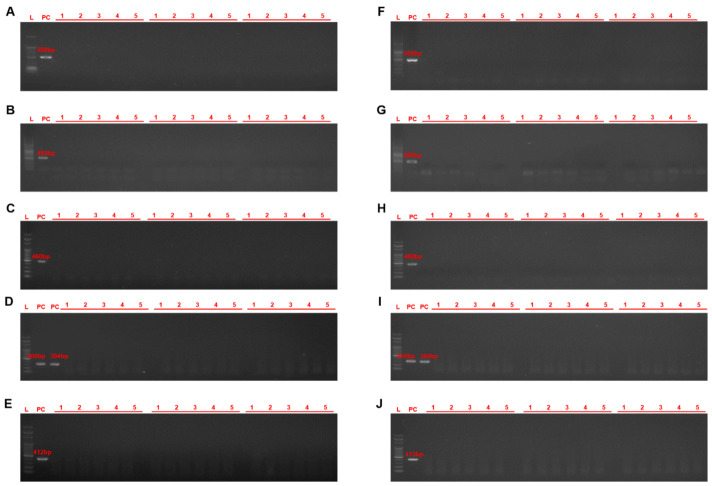
PCR amplification of *Perkinsus marinus* (**A**,**F**), *P. olseni* (**B**,**G**), *P. beihaiensis* (**C**,**H**), *Bonamia ostreae* and *B. exitiosa* (**D**,**I**), and *Marteilia refringens* (**E**,**J**) using species-specific PCR assays. L, 100 bp DNA ladder; PC, positive control; 1–5, genomic DNA from three replicate pools of six samples each of *Mytilus galloprovincialis* (**A**–**E**) and *Crassostrea gigas* (**F**–**J**).

The mussels analyzed in this study had shell heights ranging from 21.2 to 96.1 mm and tissue weights from 0.2 to 22.1 g, whereas oysters had shell lengths ranging from 15.9 to 134.2 mm and tissue weights from 0.4 to 36.3 g, indicating that multiple size classes were represented across the 18 sampling sites (Table 2 and Table 3). Species-specific PCR screening revealed no evidence of infection by regulated protozoan parasites of bivalves or by *P. beihaiensis* in any of the pooled mussel or oyster samples from port-adjacent waters on the west and south coasts of Korea: no specific amplicons were detected for *P. marinus*, *P. olseni*, *B. ostreae*, *B. exitiosa*, *M. refringens*, or *P. beihaiensis* in any field samples (Table 2 and Table 3, Figure 3). In contrast, all positive control reactions produced clear bands of the expected sizes, whereas negative controls remained free of bands, confirming that the assays were functional and that non-detection in field samples reflected a true lack of detectable infections rather than technical failure.

## 4. Discussion

In the present study, the target parasites were detected using PCR-based molecular assays. However, future studies could further improve diagnostic reliability by incorporating histological examination or microscopic observation. Molecular diagnostic methods offer high sensitivity and are particularly useful for detecting early or low-intensity infections; however, when used alone, they have limitations in determining whether the detected DNA directly reflects active tissue infection, parasite development, or associated pathological lesions [35].

These results demonstrate a complete absence of detectable infections by major protozoan pathogens of shellfish and *P. beihaiensis* in wild *M. galloprovincialis* and *C. gigas* inhabiting coastal waters near major trading ports on the west and south coasts of Korea during the 2023 survey period. This finding is notable given recent detections of *P. marinus* in wild *C. gigas* from Hongseong [25] and *B. ostreae* in *O. denselamellosa* near Gunsan Port [30], both on the west coast of Korea, and the geographical proximity of some of our sampling sites, such as Namdang and Suryong, to these previously reported locations. The absence of *P. marinus* and *Bonamia* spp. in *M. galloprovincialis* and *C. gigas* from these areas suggests that infections with these protozoan parasites may currently be focal and limited in spatial extent and may also reflect strong host specificity, as *Bonamia* spp. are known to primarily infect *Ostrea* species, with substantially lower susceptibility reported for *Crassostrea* and *Mytilus* species [34].

Thus, the failure to detect *B. ostreae* and *B. exitiosa* in the present study may reflect not only the absence or low abundance of infectious stages in the surveyed areas, but also the limited suitability of *C. gigas* and *M. galloprovincialis* as hosts. Similarly, *P. marinus* is best known as the causative agent of dermo disease in *C. virginica*, while *C. gigas* has been reported to be comparatively resistant to this parasite [36]. Differences in innate immune responses between oyster species may therefore partly explain why *P. marinus* was not detected in *C. gigas* in this survey.

The interpretation is less straightforward for *M. refringens*, as *M. galloprovincialis* has previously been reported as a susceptible host [31]. Therefore, the negative results for this parasite cannot be attributed simply to host resistance. Infection by *M. refringens* is likely influenced by several interacting factors, including infection pressure, seasonality, water temperature, salinity, and the presence of planktonic or benthic reservoirs that may contribute to its life cycle [37]. The absence of *M. refringens* in the present samples may therefore indicate that infection pressure was low at the time of sampling, that sampling did not coincide with a period of elevated transmission risk, or that the environmental and biological conditions required to maintain the parasite life cycle were not sufficiently established in the surveyed areas.

In the present study, field environmental data, including water temperature, salinity, dissolved oxygen, pH, and chlorophyll-a, were not collected at the time of sampling. This limited our ability to directly evaluate how environmental variability may have influenced the detection of the target parasites. This limitation should be considered when interpreting the present findings. In marine bivalves, the establishment, proliferation, transmission, and clinical expression of parasitic infections can be strongly influenced not only by host susceptibility, but also by water temperature, salinity, seasonality, and host physiological condition. Both *P. marinus* and *P. olseni* have been reported to show increased infection intensity and prevalence under relatively high-temperature and high-salinity conditions, particularly above 25 °C and 20 PSU [38,39,40,41,42]. In contrast, *B. ostreae* is often reported at higher prevalence from winter to spring, and parasitic protozoan stages have been shown to have reduced survival at temperatures above 25 °C and under low-salinity conditions [37,43]. For *B. exitiosa*, extreme temperatures, particularly below 7 °C or above 26 °C, as well as elevated salinity, have been suggested to influence disease dynamics [44]. In the case of *M. refringens*, a threshold temperature of approximately 17 °C has been proposed for sporulation and transmission [2], and detections of *M. refringens* have been reported more frequently during summer and autumn than during winter and spring [37]. Differences in infection intensity and prevalence under these environmental conditions would allow pathogen detection data to be interpreted beyond simple positive or negative results and would help identify environmental conditions and seasonal windows associated with elevated infection risk. Furthermore, evaluating the relationship between environmental variability and pathogen occurrence through long-term, seasonally structured monitoring could contribute to the development of early surveillance and risk prediction frameworks for non-native or regulated parasitic pathogens in Korean aquaculture areas.

Differences in local conditions or sampling seasons may partly explain the discrepancy between the present findings and previous reports from Korea [25]. Although environmental parameters were not measured at the time of sampling, the absence of PCR-positive samples across all surveyed sites, together with previous negative findings from southern coastal populations of *C. gigas* in 2020 [29], suggests that WOAH-listed protozoan parasites are not yet widely established in port-adjacent mussel and oyster populations along the surveyed coasts. However, the recent detection of *P. marinus* and *B. ostreae* in different hosts and localities in Korea indicates that the epidemiological situation may be changing. Port-adjacent waters are potential entry points for non-native pathogens because of vessel traffic, biofouling communities, ballast or exchanged seawater, and the movement of aquatic organisms. Thus, the absence of detectable infection in a single survey should not be interpreted as evidence of long-term absence or negligible risk. Future increases in seawater temperature, the movement of infected hosts, the introduction of susceptible reservoir species, or the establishment of environmental compartments involved in the transmission of *Marteilia* spp. could increase the likelihood of parasite occurrence. Although the present results indicate a low current risk in the surveyed areas, *M. refringens* should remain a priority target for surveillance in *M. galloprovincialis*, particularly under seasonal and environmental conditions that may favor transmission. Continued monitoring of multiple host species, together with measurements of temperature, salinity, seasonality, and potential reservoir organisms, will be important for improving early detection and risk assessment of protozoan parasites in Korean coastal waters.

## 5. Conclusions

This Brief Communication reports that species-specific PCR assays of 1080 *M. galloprovincialis* and 1080 *C. gigas* collected from 18 port-adjacent sites along the west and south coasts of Korea in 2023 detected no infections by *P. marinus*, *P. olseni*, *B. ostreae*, *B. exitiosa*, *M. refringens*, or *P. beihaiensis*. These data provide important baseline information on the current absence of WOAH-listed protozoan parasites in wild mussel and oyster populations inhabiting high-risk harbor environments in Korea and will support future risk assessments, disease-free certifications in shellfish trade, and the design of environment-coupled surveillance programs for transboundary shellfish diseases.

## Figures and Tables

**Table 1:** Primer sets used for species-specific PCR detection of protozoan parasites in *Mytilus galloprovincialis* and *Crassostrea gigas*.

Species	Primers	Primers Sequence (5′-3′)	Size (bp)	Target Gene	Reference
*Perkinsus marinus*	PmarITS-70F	F: CTT TTG YTW GAG WGT TGC GAG ATG	509	Inter Transcribed Spacer of rDNA gene	[32]
	PmarITS-600R	R: CGA GTT TGC GAG TAC CTC KAG AG			
*P. olseni*	HS12	F: CGA AAC TAG CGG TCT TGC TTC GGC	490	Inter Transcribed Spacer of rDNA gene	[28]
	HS13	R: AGG CGC GGT CCT CCT CKC G			
*P. beihaiensis*	PerkITS-85	F: CCG CTT TGT TTG GAT CCC	460	Inter Transcribed Spacer of rDNA gene	[33]
	PerkITS-430R	R: TCT GAG GGG CTA CAA TCA T			
*Bonamia ostreae,* *B. exitiosa*	BO	F: CAT TTA ATT GGT CGG GCC GC	300 (*B. ostreae*) 304 (*B. exitiosa*)	Small Sub Unit of rDNA gene	[34]
	BOAS	R: CTG ATC GTC TTC GAT CCC CC			
*Marteilia refringens*	Pr4	F: CCG CAC ACG TTC TTC ACT CC	412 (M type) 413 (O type)	Inter Transcribed Spacer of rDNA gene	[14]
	Pr5	R: CTC GCG AGT TTC GAC AGA CG			

**Table 2:** Infection prevalence of WOAH-listed and non-listed protozoan parasites in *Mytilus galloprovincialis* collected from the west and south coasts of Korea.

Site			N	SL (mm) Mean ± SD		TWT (g) Mean ± SD		Infection Prevalence (%)											
								*Perkinsus*						*Bonamia*				*Marteilia*	
								*marinus*		*olseni*		*beihaiensis*		*ostreae*		*exitiosa*		*refringens*	
				May	Sep.	May	Sep.	May	Sep.	May	Sep.	May	Sep.	May	Sep.	May	Sep.	May	Sep.
Western Coast	Taean Port	Eoeundol Port	60	59.9 ± 7.6	27.5 ± 3.9	4.6 ± 2.2	0.6 ± 0.2	0	0	0	0	0	0	0	0	0	0	0	0
		Tonggae Port	60	35.5 ± 7.9	60.7 ± 7.1	1.6 ± 1.2	5.6 ± 1.9	0	0	0	0	0	0	0	0	0	0	0	0
	Boryeong Port	Gungri	60	57.9 ± 3.8	62.4 ± 7.1	6.1 ± 1.4	7.0 ± 2.1	0	0	0	0	0	0	0	0	0	0	0	0
		Namdang Port	60	49.3 ± 5.2	59.0 ± 6.3	4.2 ± 1.4	6.2 ± 2.2	0	0	0	0	0	0	0	0	0	0	0	0
	Janghang Port	Bieung Port	60	67.2 ± 6.8	31.0 ± 2.9	7.3 ± 2.1	0.8 ± 0.3	0	0	0	0	0	0	0	0	0	0	0	0
	Gunsan Port	Jangja-do	60	55.1 ± 9.6	36.5 ± 6.9	5.2 ± 2.3	1.3 ± 0.8	0	0	0	0	0	0	0	0	0	0	0	0
		Gusipo Port	60	33.7 ± 2.4	63.6 ± 6.0	1.4 ± 0.3	6.7 ± 2.0	0	0	0	0	0	0	0	0	0	0	0	0
South Coast	Gwangyang Port	Gwangyang Police Station	60	59.9 ± 9.8	70.6 ± 7.1	5.9 ± 3.2	9.3 ± 3.4	0	0	0	0	0	0	0	0	0	0	0	0
		Dochon Ferry Terminal	60	50.5 ± 2.2	74.9 ± 8.3	5.4 ± 1.2	9.1 ± 2.6	0	0	0	0	0	0	0	0	0	0	0	0
		Johwa-ri	60	42.2 ± 4.9	35.3 ± 9.4	2.9 ± 1.1	1.3 ± 1.1	0	0	0	0	0	0	0	0	0	0	0	0
		Mangdeok Port	60	46.6 ± 5.8	57.8 ± 6.7	4.2 ± 1.7	3.7 ± 1.6	0	0	0	0	0	0	0	0	0	0	0	0
	Yeosu Port	Hodu-ri	60	60.7 ± 4.0	58.5 ± 6.7	5.3 ± 1.0	4.3 ± 1.5	0	0	0	0	0	0	0	0	0	0	0	0
	Tongyeong Port	Pyeongrim Port	60	49.1 ± 7.5	63.5 ± 6.7	5.3 ± 2.4	4.2 ± 1.4	0	0	0	0	0	0	0	0	0	0	0	0
		Hwasam-ri	60	50.0 ± 4.2	74.0 ± 6.2	5.1 ± 1.5	9.6 ± 2.9	0	0	0	0	0	0	0	0	0	0	0	0
	Gohyeon Port	Eogu-ri	60	46.5 ± 7.8	30.3 ± 2.7	3.8 ± 2.0	0.7 ± 0.2	0	0	0	0	0	0	0	0	0	0	0	0
		Myeongsa-Po	60	67.5 ± 5.7	55.9 ± 8.1	5.9 ± 1.5	3.8 ± 1.7	0	0	0	0	0	0	0	0	0	0	0	0
	Okpo Port	Jangmok-ri	60	65.6 ± 5.6	39.2 ± 3.6	6.5 ± 2.3	1.6 ± 0.5	0	0	0	0	0	0	0	0	0	0	0	0
	Busan Port	Angolpo	60	46.7 ± 8.3	60.8 ± 8.3	4.0 ± 2.6	5.5 ± 3.1	0	0	0	0	0	0	0	0	0	0	0	0

**Table 3:** Infection prevalence of WOAH-listed and non-listed protozoan parasites in *Crassostrea gigas* collected from the west and south coasts of Korea.

Site			N	SL (mm) Mean ± SD		TWT (g) Mean ± SD		Infection Prevalence (%)											
								*Perkinsus*						*Bonamia*				*Marteilia*	
								*marinus*		*olseni*		*beihaiensis*		*ostreae*		*exitiosa*		*refringens*	
				May	Sep.	May	Sep.	May	Sep.	May	Sep.	May	Sep.	May	Sep.	May	Sep.	May	Sep.
Western Coast	Taean Port	Mohang Port	60	56.8 ± 8.5	43.4 ± 5.9	2.5 ± 1.0	2.1 ± 1.1	0	0	0	0	0	0	0	0	0	0	0	0
		Pado-ri	60	79.1 ± 11.5	61.5 ± 6.3	6.2 ± 2.3	3.5 ± 1.5	0	0	0	0	0	0	0	0	0	0	0	0
	Boryeong Port	Gungri	60	67.8 ± 7.0	63.5 ± 12.1	3.6 ± 0.8	3.7 ± 1.6	0	0	0	0	0	0	0	0	0	0	0	0
		Suryong Port	60	49.3 ± 6.7	59.9 ± 14.8	2.0 ± 0.5	3.3 ± 1.8	0	0	0	0	0	0	0	0	0	0	0	0
	Janghang Port	Janghang Port	60	21.3 ± 4.7	45.3 ± 7.9	3.1 ± 1.5	1.0 ± 0.3	0	0	0	0	0	0	0	0	0	0	0	0
	Gunsan Port	Seonyu Port	60	53.1 ± 7.4	52.7 ± 7.1	5.4 ± 1.4	3.1 ± 0.9	0	0	0	0	0	0	0	0	0	0	0	0
		Gomso Port	60	50.0 ± 8.3	55.6 ± 9.6	3.9 ± 1.6	3.4 ± 1.2	0	0	0	0	0	0	0	0	0	0	0	0
South Coast	Gwangyang Port	Gwangyang Port	60	58.7 ± 11.0	55.1 ± 8.0	6.5 ± 2.0	2.6 ± 1.5	0	0	0	0	0	0	0	0	0	0	0	0
		Gwangyang Police Station	60	77.2 ± 11.2	40.9 ± 6.6	6.8 ± 2.7	0.9 ± 0.7	0	0	0	0	0	0	0	0	0	0	0	0
		Chonam Bridge	60	80.0 ± 14.2	54.6 ± 7.1	6.0 ± 3.5	2.9 ± 1.0	0	0	0	0	0	0	0	0	0	0	0	0
		Taein Bridge	60	73.0 ± 10.7	45.4 ± 6.5	5.4 ± 1.8	1.4 ± 0.5	0	0	0	0	0	0	0	0	0	0	0	0
	Yeosu Port	Imok-ri	60	42.0 ± 5.0	43.6 ± 6.9	1.4 ± 0.4	1.7 ± 0.5	0	0	0	0	0	0	0	0	0	0	0	0
	Tongyeong Port	Pyeongrim Port	60	66.0 ± 10.0	75.4 ± 21.3	5.8 ± 2.6	8.4 ± 7.2	0	0	0	0	0	0	0	0	0	0	0	0
		Dongam Port	60	63.8 ± 13.5	57.2 ± 7.7	4.4 ± 2.2	2.3 ± 0.6	0	0	0	0	0	0	0	0	0	0	0	0
	Gohyeon Port	Daepo Port	60	43.7 ± 5.5	47.0 ± 4.8	1.5 ± 0.6	2.3 ± 0.8	0	0	0	0	0	0	0	0	0	0	0	0
		Seondaldo Ferry Terminal	60	56.4 ± 8.8	54.4 ± 20.1	4.1 ± 1.6	3.5 ± 2.9	0	0	0	0	0	0	0	0	0	0	0	0
	Okpo Port	Jangmok-ri	60	75.0 ± 17.9	53.1 ± 13.1	13.3 ± 7.3	3.1 ± 2.2	0	0	0	0	0	0	0	0	0	0	0	0
	Busan Port	Busan New Port	60	54.4 ± 6.1	58.7 ± 7.8	2.9 ± 0.8	4.0 ± 1.3	0	0	0	0	0	0	0	0	0	0	0	0

## Data Availability

The datasets generated and analyzed during the current study were not archived in a public repository but can be obtained from the corresponding author upon reasonable request.
